# Supplementary material for: Predicting visual function by interpreting a neuronal wiring diagram
Source: Nature. 2024 Oct 2;634(8032):113–23. doi: 10.1038/s41586-024-07953-5 (PMC11446822; doi:10.1038/s41586-024-07953-5)
Supplement: Supplementary file 6 — CRF and ERF predictions for individual TmY4 and TmY9 cells. Analogous to Supplementary Data 3, but for TmY target types. Shown are the top four monosynaptic pathways, the strongest pathway passing through each of the top ten intermediary types (ranking from Extended Data Fig. 7), and the trisynaptic pathway Tm1–TmY–Dm3–TmY (see the section entitled Prediction of spatial normalization). [file 41586_2024_7953_MOESM6_ESM.zip › DataS4/TmY9qΓèÑ/720575940628566504.pdf]

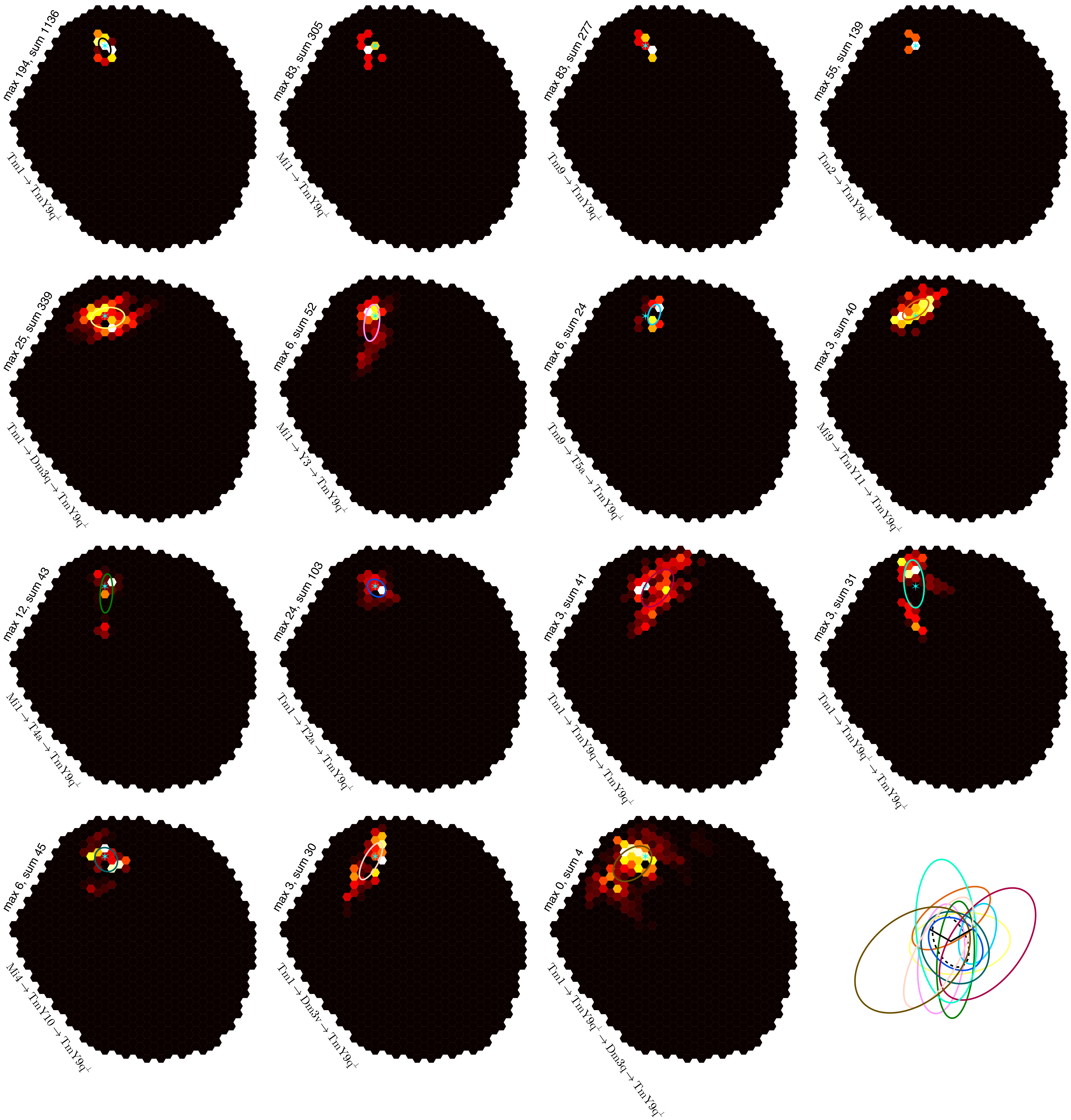

max 194, sum 1136
Tm1 → TmY9q⊥
max 83, sum 305
Mi1 → TmY9q⊥
max 83, sum 277
Tm9 → TmY9q⊥
max 55, sum 139
Tm2 → TmY9q⊥
max 25, sum 339
Tm1 → Dm3q → TmY9q⊥
max 6, sum 52
Mi1 → Y3 → TmY9q⊥
max 6, sum 24
Tm9 → T5a → TmY9q⊥
max 3, sum 40
Mi9 → TmY11 → TmY9q⊥
max 12, sum 43
Mi1 → T4a → TmY9q⊥
max 24, sum 103
Tm1 → T2a → TmY9q⊥
max 3, sum 41
Tm1 → TmY9q → TmY9q⊥
max 3, sum 31
Tm1 → TmY9q⊥ → TmY9q⊥
max 6, sum 45
Mi4 → TmY10 → TmY9q⊥
max 3, sum 30
Tm1 → Dm3v → TmY9q⊥
max 0, sum 4
Tm1 → TmY9q⊥ → Dm3q → TmY9q⊥
